# Paradoxical activation of chronic lymphocytic leukemia cells by ruxolitinib *in vitro* and *in vivo*

**DOI:** 10.3389/fonc.2023.1043694

**Published:** 2023-04-11

**Authors:** David E. Spaner, Tina YuXuan Luo, Guizhi Wang, Gideon Schreiber, Daniel Harari, Yonghong Shi

**Affiliations:** ^1^ Biology Platform, Sunnybrook Research Institute, Toronto, ON, Canada; ^2^ Department of Immunology, University of Toronto, Toronto, ON, Canada; ^3^ Department of Medical Biophysics, University of Toronto, Toronto, ON, Canada; ^4^ Department of Hematology, Sunnybrook Odette Cancer Center, Toronto, ON, Canada; ^5^ Department of Medicine, University of Toronto, Toronto, ON, Canada; ^6^ Department of Biomolecular Sciences, Weizmann Institute of Science, Rehovot, Israel

**Keywords:** Chronic lymphocytic leukemia, IL-10, cytokines, toll-like receptors, janus kinases, cancer microenvironment, ibrutinib, ruxolitinib

## Abstract

**Introduction:**

Chronic lymphocytic leukemia (CLL) is characterized by an aberrant cytokine network that can support tumor growth by triggering janus kinase (JAK)/STAT pathways. Targeting cytokine-signaling should then be a rational therapeutic strategy but the JAK inhibitor ruxolitinib failed to control and seemingly accelerated the disease in clinical trials.

**Methods:**

The effect of ruxolitinib on primary human CLL cells was studied *in vitro* and *in vivo*.

**Results:**

Ruxolitinib increased phosphorylation of IRAK4, an important toll-like receptor (TLR)- signaling intermediate, in circulating CLL cells *in vitro*. It also enhanced p38 and NFKB1 phosphorylation while lowering STAT3 phosphorylation in CLL cells activated with TLR-7/8 agonists and IL-2. Among the cytokines made by activated CLL cells, high levels of IL-10 contributed strongly to STAT3 phosphorylation and inhibited TLR7 activity. Ruxolitinib limited TLR-mediated *IL10* transcription and markedly reduced IL-10 production *in vitro*. It also decreased blood levels of IL-10 while increasing TNFα along with phospho-p38 expression and gene sets associated with TLR-activation in CLL cells *in vivo*. The bruton's tyrosine kinase inhibitor ibrutinib decreased IL-10 production *in vitro* but, in contrast to ruxolitinib, blocked initial *IL10* transcription induced by TLR-signaling in vitro, decreased TNFα production, and deactivates CLL cells *in vivo*.

**Discussion:**

These findings suggest the possible benefits of inhibiting growth factors with JAK inhibitors in CLL are outweighed by negative effects on potential tumor suppressors such as IL-10 that allow unrestrained activation of NFκB by drivers such as TLRs. Specific inhibition of growth-promoting cytokines with blocking antibodies or infusing suppressive cytokines like IL-10 might be better strategies to manipulate cytokines in CLL.

## Introduction

Inhibitors of bruton’s tyrosine kinase (BTK) such as ibrutinib or of BCL-2 like venetoclax and chimeric antigen receptor (CAR)-T cells have increased treatment options and improved outcomes for CLL patients (1–3). Unfortunately, these novel modalities are rarely curative as single agents and patients with CLL symptomatic enough to require treatment are still likely to die eventually of their disease. Additional therapeutic targets are needed to provide further lines of therapy and overcome resistance to existing agents.

Aberrant cytokine-signaling would seem to be a rational drug target in CLL. Cytokines such as IL-10, IL-6, IL-4, interferon (IFN)-β, and IFN-α signal through combinations of janus kinases (JAKs) to phosphorylate signal transduction and activator of transcription (STAT) proteins (4). Members of this family such as STAT3 regulate genes that promote the growth and survival of CLL cells (5, 6).

Cytokines can also mediate drug-resistance. Growth and anti-apoptotic signals from the B cell receptor (BCR) along with chemokine- and toll-like-receptors (TLRs) are delivered to CLL cells in lymphoid organ microenvironments called proliferation centers (PCs) through pathways that involve BTK and are blocked by ibrutinib (7). BTK-independent microenvironmental signals may allow CLL cells to persist in the presence of ibrutinib (8). We showed IFN-signaling remains active in patients on ibrutinib and may support survival of CLL cells and eventual disease progression (9). Cytokine-signaling may also cause resistance to cytotoxic drugs like venetoclax by upregulating anti-apoptotic BCL-2 family members such as MCL1 (10). STAT3 is a central mediator of immunosuppression in the cancer microenvironment and its activation by cytokines, particularly IL-10, could mediate resistance to CAR-T cells (11, 12).

Based on these considerations, we carried out several clinical trials with ruxolitinib in CLL patients (13–15). Ruxolitinib is a JAK1/2 inhibitor approved for the treatment of graft-versus-host disease and myeloid disorders including myelofibrosis and polycythemia vera (15). By blocking growth-promoting effects of cytokines, ruxolitinib was expected to behave much like ibrutinib as a single agent but it turned out to have dramatically opposite clinical activity (13). Like ibrutinib, ruxolitinib did cause an initial lymphocytosis with decreased lymphadenopathy, thought to reflect trafficking of leukemia cells from PCs into the blood (7, 13). With ibrutinib, lymphocytosis, lymphadenopathy, and serum lactate dehydrogenase (LDH) levels that reflect metabolic activity (16) characteristically decrease over time (1). In contrast, sustained lymphocytosis, recurrent lymphadenopathy, and increased LDH levels that accompany more aggressive clinical behavior were seen with ruxolitinib (13). Ibrutinib generally improves anemia and gives prolonged disease control (1) while ruxolitinib caused significant anemia, potentially from increases in TNFα that can suppress erythropoiesis (17), and had little therapeutic activity during the time it was administered to patients (13). Similar but more muted effects were observed when ruxolitinib was given to patients on ibrutinib with lower tumor burdens (14, 15).

Taken together, these observations suggested CLL cells become activated when cytokine-signaling was blocked by ruxolitinib *in vivo*. The studies in this paper were undertaken to try to identify possible mechanisms for this effect.

## Methods

### Antibodies and reagents

Fluorescent CD83 and TNFα antibodies were obtained from BD Biosciences (Bedford, MA, USA). Human IL-10, IL10R, and IL-6 blocking antibodies were from eBioscience (San Diego, CA, USA). Resiquimod and β-actin antibodies were from Sigma-Aldrich (St Louis, MO, USA). Ruxolitinib was from SelleckChem (Houston, TX, USA). The IL-6 receptor-blocking antibody Actemra (Roche Canada, Mississauga, ON, Canada), IL-2 (Chiron, Corp., San Francisco, CA, USA), and human IFN-β1b (Novartis Pharmaceuticals Canada Inc, Dorval, QC, Canada) were purchased from the Sunnybrook Cancer Centre pharmacy. IL-10, IL-6, IL-4, CXCL10, CXCL8, CCL2, CLL3, and CCL5 were from Peprotech (Rocky Hill, NJ, USA). The TACE inhibitor, TAPI, was from Peptides International (Louisville, KY). AIM-V serum-free media was from Thermofisher Scientific (Mississauga, ON, Canada). Goat anti-human IgM Fc-specific antibodies were from Jackson ImmunoResearch Labs (West Grove, PA, USA). Phospho-(Y705) STAT3 (Cat. No 9131), total STAT3, phospho-NFκB p105 (Ser933) (Cat. No. 4806), total p105, phospho-p38 MAPK (Thr180/Tyr182) (Cat. No. 9211), total p38, phospho-IRAK4 (Thr345/Ser346) (Cat.No 11927), total IRAK4, and secondary horseradish peroxidase-conjugated anti-rabbit and anti-mouse antibodies (Cat. Nos. 7074 and 7076, respectively) were from Cell Signaling Technology (Beverly, MA). The IFN-α receptor (IFNAR) antibody anifrolumab (18) was a gift from AstraZeneca.

### CLL cells

For most *in vitro* experiments, CLL cells were isolated as before (6, 9, 10) by negative selection from the blood of consenting patients attending a specialized CLL clinic at Sunnybrook. Cells were used immediately and patients were untreated for at least 6 months prior to blood collection.

In some instances, CLL cells and plasma stored at -80°C from a previously described trial of ruxolitinib in symptomatic CLL patients (ClinicalTrials.gov, NCT02015208) (13) were used for immunoblotting and cytokine measurements.

### Cell culture

Purified CLL cells (1.5x10^6^ cells/ml) were cultured in serum-free AIM-V medium plus 2-ME (Sigma-Aldrich) (5x10^−5^ M) in 6- or 24-well plates (BD Labware) at 37°C in 5% CO_2_ for the times indicated in the figure legends. Resiquimod and IL-2 were used at 1 μg/ml and 500 U/ml, respectively. CLL cells stimulated with IL-2 and Resiquimod are designated “2S” cells (19, 20).

Co-culture experiments on OP9 control and CD40L-expressing OP9 stromal cells were performed as described before (20). Stromal cells were seeded into 24-well plates at a concentration of 5x10^4^ cells/well 24 h prior to initiation of CLL co-cultures and incubated at 37°C and 5% CO_2_ in alpha-MEM supplemented with 20% FBS (Gibco). After confirming the stromal layer was confluent by phase contrast microscopy, CLL cells were added at a final concentration of 2-6x10^6^ cells/ml along with IL-4 (10 ng/ml) in the presence or absence of ruxolitinib. Culture supernatants were collected 48 h later.

### Immunophenotyping

Staining of nucleated cells was determined as before (9, 19) by gating on forward- and side-scatter properties with isotype-matched irrelevant antibodies (PharMingen) as negative controls. Ten thousand viable counts were analyzed with a FACScan flow cytometer that was standardized with SpheroParticles (Spherotech Inc., Chicago, IL) and CELLQUEST software (Becton Dickinson, San Jose, CA).

### Membrane TNFα detection

Ten million CLL cells were cultured with or without resiquimod in 5-mL polystyrene tubes (Becton Dickinson Labware). TAPI (100 μmol/L), followed by CD83-FITC and TNFα-PE antibodies 4 h later, were added to each tube as before (21). Subsequent steps paralleled conventional immunophenotyping.

### Cytokine measurements

IL-10, IL-4, IL-6, IFN-α, and TNFα in plasma samples and culture supernatants were measured by Multiplexing LASER Bead Technology as a commercial service by Eve Technologies (Calgary, AB, Canada) using the Human Cytokine/Chemokine 48-Plex Discovery Assay® Array (HD48) and Human Cytokine Proinflammatory Focused 15-Plex Discovery Assay® Array (HDF15) as before (10, 13, 14). Concentrations were determined from standard curves. Assays were linear between 30 and 1000 pg/mL of cytokine.

### Immunoblotting

Protein extraction and immunoblotting were performed as before (10). Proteins were resolved in 10% sodium dodecyl sulfate-polyacrylamide gel electrophoresis and transferred to Immobilon-P transfer membranes (Millipore Corp., Billerica, MA). Western blot analysis was performed according to the manufacturers’ protocols for each antibody. Chemiluminescent signals were created with SupersignalWest Pico Luminal Enhancer and Stable Peroxide Solution (Pierce, Rockford, IL) and detected with a Syngene InGenius system (Syngene, Cambridge, United Kingdom). For additional signal, blots were stripped for 60 min at 37°C in Restore Western Blot stripping buffer (Pierce), washed twice in Tris-buffered saline plus 0.05% Tween-20 at room temperature, and reprobed as required. Densitometry was performed using Image J software. The densitometry value for each sample was normalized against the value for β-actin to obtain the intensities for phosphorylated- and total STAT3, -p105, -p38, and -IRAK4 in the figures.

### Real-time PCR

RNA was prepared with the RNeasy mini kit (Qiagen, Valencia, CA, USA), and cDNA synthesized from 2 μg of RNA using Superscript III reverse transcriptase (Life technologies, Invitrogen), according to the manufacturer’s instructions. *IL10* and hypoxanthineguanine phosphoribosyl transferase (HPRT) transcripts were amplified with the following primers:

**Table:** 

Gene	Forward	Reverse
*IL10*	5′- CATCGATTTCTTCCCTGTGA -3′	5′- CGTATCTTCATTGTCATGTAGGC -3′
*HPRT*	5’- GAGGATTTGGAAAGGGTGTT -3’	5’- ACAATAGCTCTTCAGTCTGA -3’

Polymerase chain reactions were performed in a DNA engine Opticon System (MJ Research, Waltham, MA, USA) and cycled 34 times after initial denaturation (95°C, 15 min) with the following parameters: denaturation at 94°C for 20 sec; annealing of primers at 58°C for 20 sec, and extension at 72°C for 20 sec. Abundance of transcripts was evaluated by a standard amplification curve relating initial copy number to cycle number. Copy numbers were determined from two independent cDNA preparations for each sample. The final result was expressed as fold change of target gene relative to HPRT.

### RNASeq

RNA extracted from CLL cells purified from 6 patients before taking ruxolitinib and again while on ruxolitinib for either 4 or 8 weeks was subjected to the PCR-based Ampliseq Transcriptome Human Assay using a Thermofisher ion S5xl instrument. The AmpliseqRNA plug-in Ion-torrent server was used to provide initial read numbers per gene and normalization for all 10 samples. From an initial list of 20812 genes, 12477 remained after filtering out low-expressed genes (less than 10 reads in 10 samples) and the large class of olfactory receptors (22) prior to GSEA analysis. RNAseq data is deposited at Gene Expression Omnibus (GEO) as dataset GSE197811.

### Gene set enrichment analysis

Control and experimental groups (eg. samples without ruxolitinib versus samples with ruxolitinib) were compared by the methods of GSEA (version 4.1.0, Broad Institute) (23, 24). Enrichments were considered significant with a false discovery rate (FDR)<5% and nominal p-value<1%.

### Statistical analysis

Comparisons between two groups of measurements were tested for significance by ANOVA and Student’s or paired t-tests with p<0.05 considered significant.

### Study approval

All studies involving human samples were reviewed and approved by the Sunnybrook Research Ethics Board (PIN 222-2014).

## Results

### Effect of ruxolitinib *in vivo*

**Figure 1:**
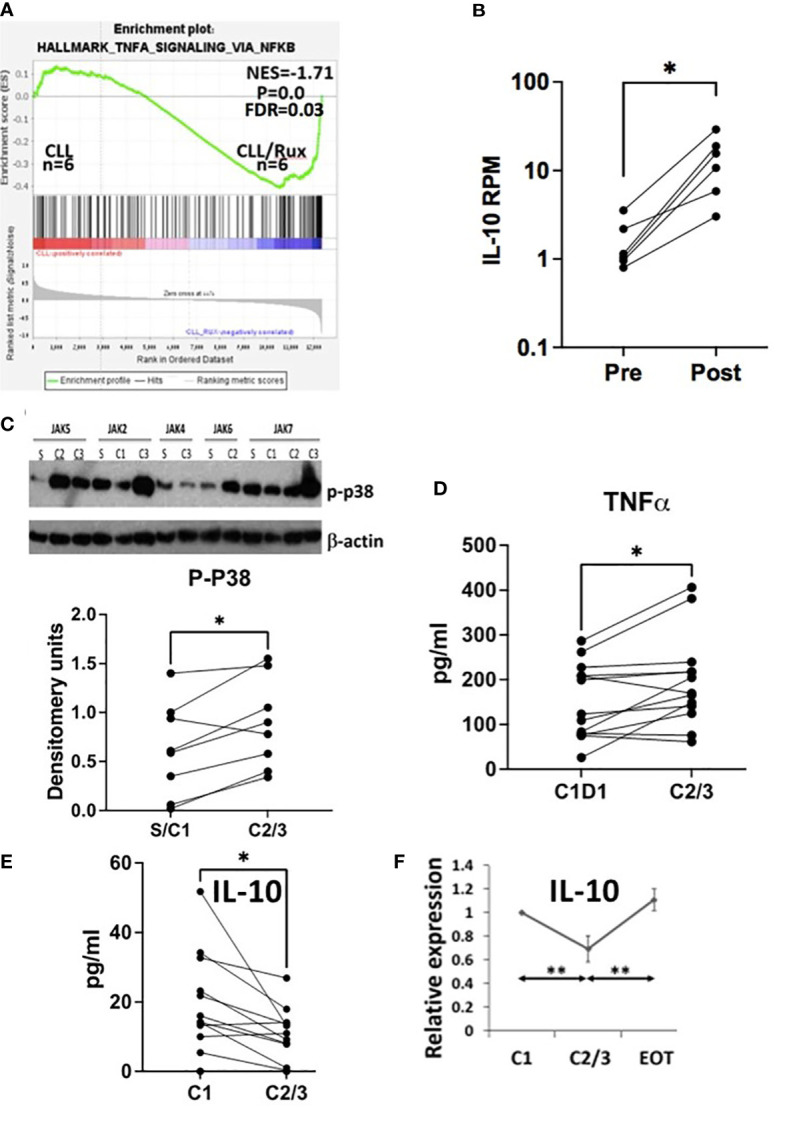
Effect of ruxolitinib *in vivo*. **(A)** Differentially expressed genes in CLL cells purified before and during administration of ruxolitinib were analyzed by GSEA. The enrichment plot indicates CLL cells exposed to ruxolitinib *in vivo* resemble immune cells that have turned up NFκB-activated genes. **(B)** Number of reads mapped per gene per million reads mapped (RPM) for *IL10* are shown in CLL cells isolated from the trial patients before (pre) and while on ruxolitinib (post) with each line representing a single patient. **(C)** Protein extracts were made from purified circulating CLL cells at the indicated times after administration of ruxolitinib (S=screening visit; C1=prior to cycle 1; C2=prior to cycle 2; etc.). Phospho-p38 was measured by immunoblotting and densitometry and normalized to β-actin. An example of an immunoblot is shown (top) and the bottom graph represents densitometry measurements for p-p38 with each line representing an individual patient. D-F. TNFα **(D)** and IL-10 **(E, F)** were measured in plasma collected from 13 patients at C1 prior to taking ruxolitinib and again either 4 (C2) or 8 weeks (C3) later. Each line represents results for an individual patient. **(F)** IL-10 at each time-point was normalized to the C1 value and shown as the average and standard error of the relative changes for all patients. EOT=end of treatment or 4 weeks after discontinuation of ruxolitinib. *, p<.05; **, p<.01. Statistical analysis was done with students paired t tests.

GSEA analysis of RNAseq data from 6 patients before and after taking ruxolitinib as a single agent for 4 or 8 weeks (13) suggested CLL cells exhibited much stronger NFκB activity in the presence of ruxolitinib (**Figure 1A**). *IL10* mRNA expression was increased in CLL cells exposed to ruxolitinib *in vivo* (**Figure 1B**) and expression of core enrichment genes including *CD83* and *SOCS3* for the “Hallmark_TNFA_Signaling_via_NFκB” data set (23, 24) is shown in **Supplementary Figure 1A**. Ruxolitinib has been shown to decrease pSTAT3 levels in CLL cells *in vivo* (13) and GSEA analysis suggested it also down-regulated type 1 IFN signature genes (**Supplementary Figure 1B**), consistent with inhibition of JAK-signaling.

Consistent with activation of TNF-signaling (21, 25), phospho-p38 increased in CLL cells exposed to ruxolitinib *in vivo* in 8 evaluable patients (**Figure 1C**). Plasma levels of TNFα also increased in CLL patients treated with ruxolitinib for 4 (C2) or 8 (C3) weeks (**Figure 1D**) while IL-10 levels decreased (**Figure 1E**). Note that CLL cells in the blood were increased from baseline at these times (13), suggesting this latter observation was not simply due to fewer IL10-producing leukemia cells (26). Plasma IL-10 after 2-3 cycles of treatment (C2/3) and one month after stopping ruxolitinib (EOT) were normalized to initial values at C1 and the relative ratios averaged for 7 evaluable patients. The results indicated IL-10 returned to pretreatment levels within a month of stopping ruxolitinib (**Figure 1F**).

### Activation of CLL cells by ruxolitinib *in vitro*

In an attempt to understand how ruxolitinib turned up NFκB-regulated genes and plasma TNFα proteins while decreasing phospho-STAT3 (13), IFN-stimulated genes, and plasma IL-10 *in vivo* (**Figure 1** and **Supplementary Figure 1**), CLL cells were purified from blood and cultured as described previously on OP9 stromal cells or OP9 cells engineered to express CD40L along with IL-4 (20) in the presence of absence of ruxolitinib at 500 nM, a dose that approximates therapeutic plasma concentrations (27). Cytokines in the culture supernatants were then measured after 48 h. In this commonly used model of the CLL microenvironment (20), TNFα was decreased significantly by ruxolitinib with inconsistent effects on IL-10 (**Supplementary Figure 2**). These cytokine changes appeared to be the opposite of what had been observed *in vivo* (**Figure 1**) and suggested invoking another microenvironmental model.

**Figure 2:**
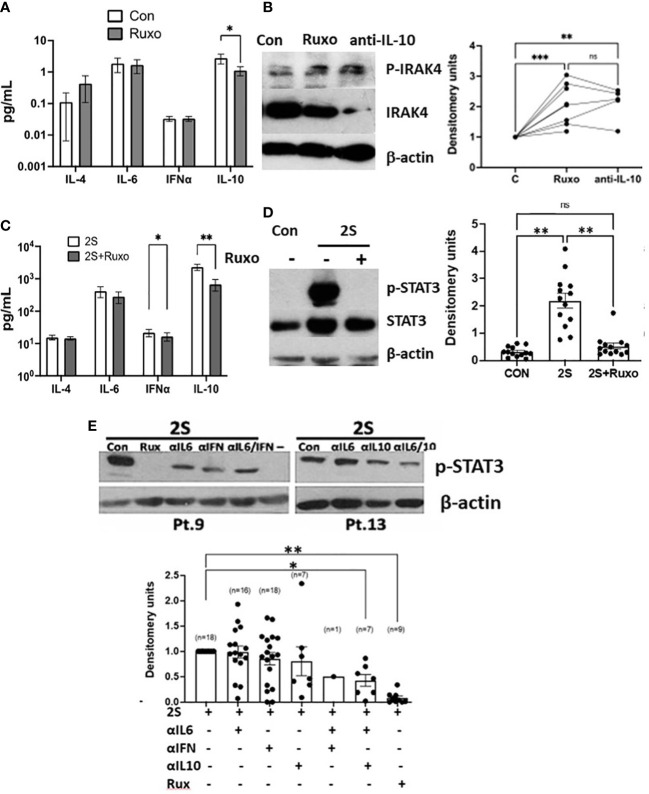
Divergent IRAK4 and STAT3 activity in ruxolitinib-treated CLL cells *in vitro*. CLL cells were cultured in AIM-V with (2S) or without (Con) IL-2 (500 U/ml) and resiquimod (1 μg/ml) and with or without ruxolitinib (Ruxo) (500 nM) or combinations of antibodies against IL-10, the IL-6 receptor, and the type 1 IFN receptor (all at 10 ng/ml). **(A, C)** Cytokines were measured in culture supernatants after 48 h from 13 **(A)** and 19 **(C)** individual patient samples. **(B, D, E)** After 18 h, phospho-IRAK4 **(B)** and -STAT3 **(C, D)** were measured by immunoblotting and densitometry and normalized to β-actin. Examples of immunoblots are shown. **(E)** The summary graph represents relative pSTAT3 levels obtained by dividing densitometry measurements in the cytokine inhibitor combinations by the measurement in control cells. Lines and closed circles in the graphs represent densitometry measurements for individual patient samples. Averages and standard errors are indicated by the boxes. *, p<.05; **, p<.01; ***, p<.025. Statistical analysis was done by 2-way ANOVA.

To identify signaling pathways that might be activated by JAK inhibition or when IL-10 levels were lowered, CLL cells were cultured directly in ruxolitinib or IL-10 antibodies. Note that ruxolitinib did not decrease the viability of CLL cells after 48 h (**Supplementary Figure 3**). The magnitude of spontaneous IL-10 production by cultured CLL cells exhibited inter-patient variability (**Supplementary Figure 4A**) but was reduced significantly by ruxolitinib (**Figure 2A**). Increased phospho-IRAK4 levels and down-regulation of total IRAK4 were apparent in a number of samples (**Figure 2B**). IRAK4 links the myddosome to down-stream activation of p38, NFκB, and STAT3 and is central to signal transmission through IL-1 and TLRs (28, 29). Given the potential role of TLRs in CLL pathobiology (30, 31) and well-known regulation of TLR-signaling by IL-10 (25), this observation suggested ruxolitinib might enhance TLR-signaling responses in CLL cells.

### Effect of ruxolitinib on cytokine production and STAT3-phosphorylation in TLR-activated CLL cells

Examination of a public database suggested CLL cells increase *IL10* mRNA expression following entry into PCs where they receive BCR- and TLR-signals that activate NFκB (**Supplementary Figure 5**) (30, 31). CLL cells with germline unmutated (U) *IGHV* genes generally behave in a more aggressive manner and are less sensitive to cytotoxic drugs than cells with mutated (M) (≤98% germline) *IGHV* genes (26, 32). A trend to increased *IL10* expression in lymph nodes was seen in M-CLL cells but the difference was not significant (**Supplementary Figure 5B**).

Since BCR-signaling is reported to induce IL-10 production by CLL cells (33), IL-10 production following cross-linking of the BCR with IgM antibodies was compared to stimulation by IL-2 and the TLR7/8 agonist resiquimod (called “2S” cells). IL-10 production following BCR-cross-linking was much lower (**Supplementary Figure 4B**) despite effective activation evidenced by increased IL-8 production (**Supplementary Figure 6A**). No significant differences in autocrine IL-10 production were noted between U- and M-CLL cells following stimulation with resiquimod with or without IL-2 (**Supplementary Figures 4B, 6B**).

Based on these considerations, ruxolitinib was studied in the 2S-model where some of the microenvironmental signals in PCs are mimicked by using IL-2 as a representative T cell factor along with resiquimod (19, 20). Activation of TLR-7 phosphorylates IRAK4 leading to phosphorylation of p38 and p105 (NFKB1), an intermediate of the NFκB pathway (34), leading to induction of cytokines that can signal in an autocrine and paracrine fashion *via* STAT3 (25, 35). In addition to exogenous IL-2 in the 2S cultures, IL-10, IFN, IL-6, and IL-4 can potentially tyrosine-phosphorylate STAT3 (6, 34, 36, 37). These cytokines were measured in supernatants of CLL cells cultured with IL-2 and resiquimod for 48 h (**Figure 2C** and **Supplementary Figure 4A**). IL-4, IL-6, and IFN-α were made spontaneously at low levels, if at all, by CLL cells in the absence of exogenous stimulation (**Figure 2A**). They were generally increased by IL-2 and resiquimod but only to the order of ~ 10 pg/ml for IL-4 and IFN-α and ~100 pg/ml for IL-6. Ruxolitinib generally decreased IFN-α levels but had variable effects on IL-4 and IL-6 (**Figures 2A, C** and **Supplementary Figure 4A**). In contrast, spontaneous IL-10 production by CLL samples reached the order of 1000 pg/ml following stimulation with IL-2 and resiquimod and was strikingly reduced by ruxolitinib, particularly in TLR-activated cells (**Figure 2C** and **Supplementary Figure 4A**).

Remarkably, the increases in phospho-STAT3 levels caused by IL-2 and resiquimod after 24 h were almost entirely prevented by ruxolitinib (**Figure 2D**).

### Cytokine contributions to pSTAT3 expression in activated CLL cells

Blocking antibodies were used to assess how the individual cytokines affected phospho-STAT3 expression in 2S CLL cells (**Figure 2E**). IL-10 antibodies proved most effective at lowering pSTAT3 levels in these conditions and had additive effects with IL-6 antibodies but not to the extent of non-specific cytokine-signaling blockade with ruxolitinib (**Figure 2E**). Note that a combination of antibodies to IL-10 and IL-6 was shown previously to prevent STAT3 phosphorylation in CLL cells activated by resiquimod without IL-2 (38). Taken together, these findings suggested STAT3 was phosphorylated by a number of cytokines with major contributions from IL-2 and -10.

### Phenotype of TLR-activated CLL cells in the presence of ruxolitinib *in vitro*

**Figure 3:**
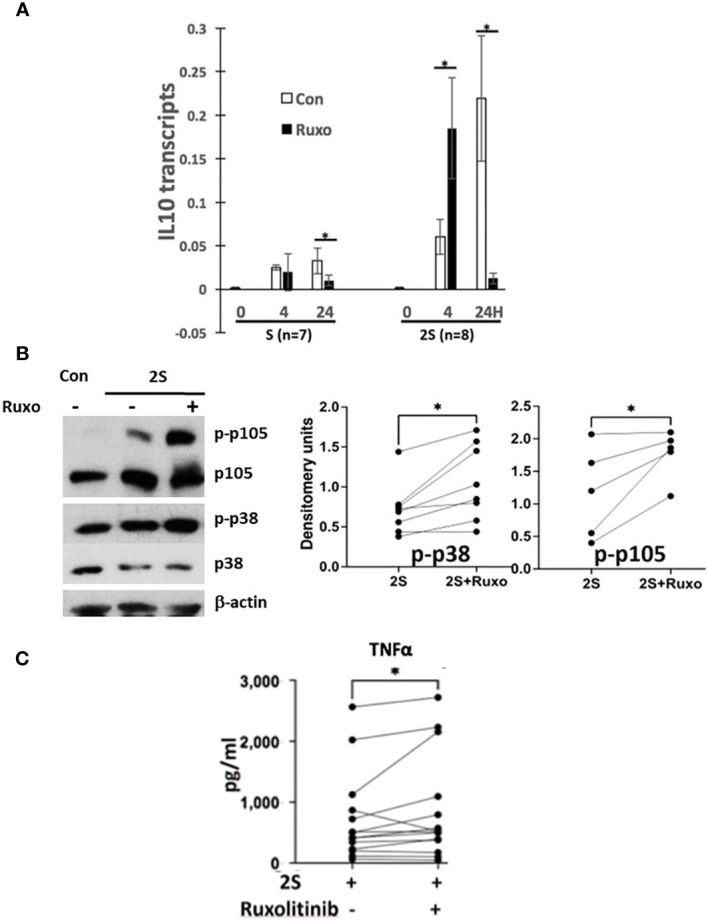
Effect of ruxolitinib on *IL10* transcription, p38 and NFκB-signaling and TNFα production by activated CLL cells. Purified CLL cells were cultured in AIM-V alone (Con), with resiquimod (S28) (1 μg/ml), or with IL-2 (500 U/ml) and resiquimod (2S) in the presence or absence of ruxolitinib (Ruxo) (500 nM). **(A)** *IL10* transcripts were measured after 4 and 24 h by quantitative RT-PCR. **(B)** Phospho-p38 and -p105 were measured by immunoblotting and densitometry and normalized to β-actin. Examples of immunoblots are shown. **(C)** TNFα in culture supernatants was measured after 48 h. Lines and dots in the graphs represent results for individual patient samples. *, p<.05. Statistical analysis was done with students paired t tests and 2-way ANOVA.

CLL cells from patients on ruxolitinib were exposed to higher TNFα and lower IL10 plasma levels and exhibited an activation phenotype consisting of increased expression of NFκB-regulated genes including *IL10* and *CD83* along with phosphorylated intermediates of NFκB-signaling pathways coupled with lower phospho-STAT proteins (**Figure 1** and **Supplementary Figure 1**). The *in vivo* effects of ruxolitinib on IL-10 and pSTAT3 levels were reproduced in the 2S model (**Figure 2**). To determine if ruxolitinib affected the other activation markers, *IL10* transcription in CLL cells in response to resiquimod with (2S) or without (S) IL-2 was measured at 4 and 24 h (**Figure 3A**). Resiquimod caused an early increase in *IL10* transcript levels at 4 h that was sustained at 24 h (**Figure 3A**, left side of graph) and amplified considerably by IL-2 (**Figure 3A**, right side of graph). The increase in *IL10* transcripts that had been seen *in vivo* with ruxolitinib (**Figure 1B**) was also observed in 2S cells at 4 h. Interestingly, ruxolitinib reduced *IL10* expression significantly at 24 h in both activation conditions (**Figure 3A**).

Consistent with enhanced TLR-signaling, ruxolitinib increased phospho-p38 and -p105 levels in CLL cells activated by resiquimod and IL-2 (**Figure 3B**). Enhanced TLR-signaling was also suggested by higher TNFα levels in the presence of ruxolitinib (**Figure 3C**) as TNFα production by 2S cells is mainly from the TLR agonist (35). However, the effect of ruxolitinib on TNFα was small and mainly from a subset of the samples (**Figure 3C**). Accordingly, the results were reclassified on the basis of the mutational status of *IGHV* and p53 genes in the samples (**Supplementary Figure 4C**). Interestingly, TNFα production was significantly higher from M-CLL samples (n= 11) but not changed significantly and often even decreased in U-CLL cells (n=8). Too few mutant p53 samples (n=2) were available to draw conclusions.

### Cytokine-mediated inhibition of TLR-signaling in CLL cells

**Figure 4:**
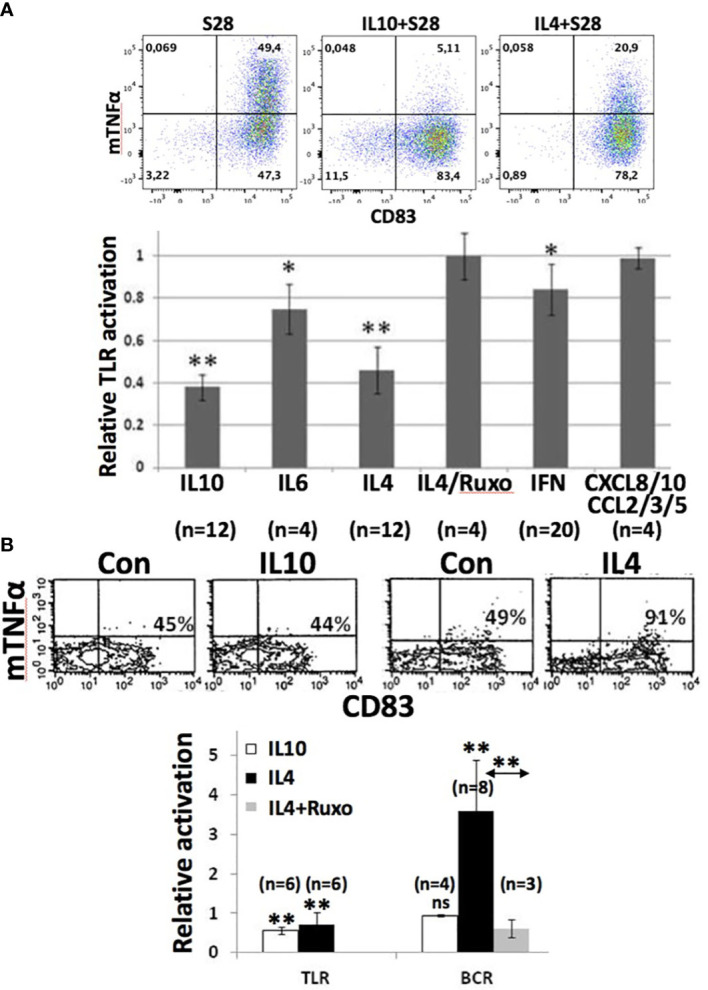
Effect of cytokines and chemokines on TLR- and BCR-signaling in CLL cells *in vitro*. CLL cells were cultured for 48 h in the presence or absence of pre-optimized concentrations of IL-10 (10 ng/ml), IL-6 (100 ng/ml), IL-4 (40 ng/ml), IFNβ (500 U/ml), or a mixture of CXCL8, CXCL10, CCL2, CCL3, CCL5 (each at 20 ng/ml) with or without ruxolitinib. The cells were then stimulated with resiquimod **(A)** or anti-IgM antibodies **(B)** and membrane TNFα and CD83 expression measured 4 h later by flow cytometry. Examples are shown above the summary graphs. Numbers in the histograms represent percentages of mTNFα^+^CD83^+^ cells for TLR-activation and CD83^+^ cells for BCR-crosslinking. Relative TLR- or BCR-activation were determined for each sample by dividing the percentage of mTNFα^+^CD83^+^ cells or CD83^+^ cells, respectively, following co-culture with cytokines by the percentages obtained in control cultures. BCR-activation with IL4 plus ruxolitinib is reported relative to the result with IL4. The average and standard error of relative TLR- or BCR-activation for the indicated numbers of patient samples are shown in the graphs. *, p<.05; **, p<.01.

The relationship of increased NFκB activation with decreased STAT3 phosphorylation caused by ruxolitinib (**Figure 2D**) suggested JAK-activating cytokines in the cultures might be inhibiting TLR-signaling in CLL cells. IL-2 promotes TLR-signaling in CLL cells in part by increasing MAPK pathway activity (35) but the other cytokines can suppress TLR-responses under certain conditions. For example, TLR-mediated production of TNFα by CLL cells can be inhibited by prior treatment with IL-6 and restored by a JAK inhibitor (34) and IL-4 can block NFκB at the DNA level (39). To determine how these cytokines affected resiquimod-signaling in CLL cells *in vitro*, purified CLL cells were cultured for 1-2 days in pre-optimized concentrations of IL-10, IL-6, IL-4, IFN-β or a mixture of the chemokines CXCL8, CXCL10, CCL2, CLL3, and CCL5 some of which can also activate STAT3 (40, 41). To assess effects on TLR-signaling, surface levels of TNFα (mTNFα) were measured 4 h after stimulation with resiquimod as before (21, 34). Relative TLR-activation was calculated by dividing the percentage of mTNFα^+^ cells after pre-culture in the cytokines and chemokines by the percentage obtained after pre-culture in serum-free AIM-V media alone. Prior exposure to IL-10 consistently inhibited TLR-responses in this assay (**Figure 4A**). IL-4 was also strongly inhibitory and the inhibition could be reversed by ruxolitinib (**Figure 4A**). IFN-β inhibited TLR-responses in many patient samples while IL-6 was the least inhibitory cytokine and the chemokines did not significantly affect TLR-signaling (**Figure 4A**).

### Effect of IL-10 and -4 on BCR-signaling

The suppressive effects of IL-10 and IL-4 (**Figure 4A**) on TLR-signaling might be relieved by ruxolitinib to account for its ability to activate CLL cells *in vivo*. To distinguish between these cytokines, their effects on BCR-signaling were compared, as BCR-signaling is considered the major driver of CLL progression and known to be regulated by IL-4 (42, 43). CLL cells were cross-linked with IgM antibodies and CD83 measured on the cell-surface by flow cytometry 4 h later. CD83 was used as an NFκB-reporter gene (**Supplementary Figure 1A**) because TNFα expression was lower after BCR-activation than after TLR-activation (**Figure 4B**). Relative BCR-activation was then calculated as the percentage of CD83^+^ CLL cells in response to IgM-cross-linking after 48 h in IL-10 or IL-4 divided by the percentage of CLL cells cultured only in serum-free media prior to IgM-cross-linking. IL-10 did not affect BCR-signaling significantly (**Figure 4B**) in this assay. In contrast, IL-4 enhanced BCR-signaling responses significantly (**Figure 4B**, right dot plots and summarized in the bottom graph). The promoting effect of IL-4 on BCR-signaling was reversed by ruxolitinib (**Figure 4B**, bottom graph).

### Effect of IL-10 on IL10 transcription in resting and activated CLL cells

Ruxolitinib blocks JAK-mediated signals and significantly inhibited “late” *IL10* transcription at 24 h but did not affect resiquimod-induced transcription at 4 h and even enhanced it in the presence of IL-2 (**Figure 3A**). Given the major decline of IL-10 levels in culture supernatants caused by ruxolitinib (**Figures 2A, C**), this result suggested the importance of sustained *IL10* transcription for protein production.

**Figure 5:**
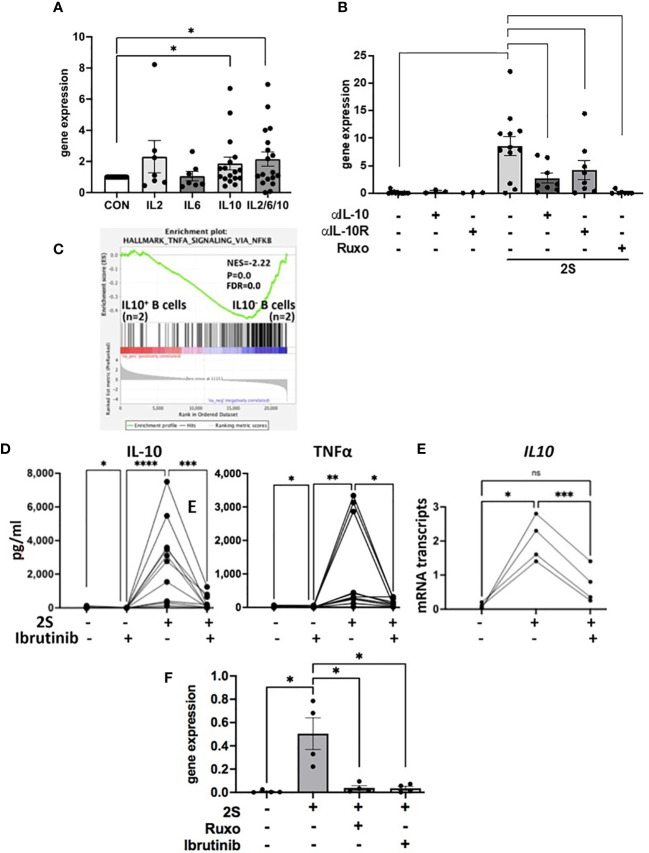
Regulation of *IL10* transcription and TLR-activation in the presence of ibrutinib. CLL cells were cultured alone, with IL-2, IL-6, IL-10, or combinations of IL-2, -6, and -10 **(A)** or with IL-2 and resiquimod (2S) in the presence and absence of IL-10 or IL-10 receptor blocking antibodies **(B)**, ruxolitinib **(B, F)**, or ibrutinib (300 nM). **(A, B, E, F)** *IL10* transcripts were measured after 4 **(E)** and 24 h **(A, B, F)**. **(D)** IL-10 (left panel) and TNFα (right panel) in culture supernatants were measured after 48 h. Averages and standard errors are shown in the boxes and the lines and closed circles represent results for individual samples. **(C)** Microarray data from 2 samples each of IL-10-secreting and -nonsecreting CD69^+^ human B cells indicated by an IL-10 capture assay after 2 days of stimulation with CpG oligonucleotides, IL-4, and CD40 antibodies were down-loaded from data-set GSE49853. DEGs were obtained with GEO2R software, ranked in order of decreasing logFc values, and analyzed by GSEA against the Hallmarks gene set collection in MsigDB. The plot depicts significant enrichment of NFκB-signaling genes in TLR-activated human B cells in the absence of autocrine IL10. *, p<.05;**, p<.01; ***, p<.001; ****, p<.0001; ns=not significant. Statistical analysis was done by students paired t tests and 2-way ANOVA.

In human macrophages, IFN-β induced by the TLR-4 agonist LPS is required to sustain *IL10* transcription (44) and IFN-signaling is blocked by ruxolitinib (9). To address the possibility that a resiquimod-induced autocrine IFN-loop sustains *IL10* transcription and translation, IL-10 levels in culture supernatants were measured 48 h after activating CLL cells with resiquimod (S) or resiquimod and IL-2 (2S) in the presence or absence of the IFNAR antibody anifrolumab (18). In contrast to macrophages and independent of IL-2, anifrolumab did not markedly decrease IL-10 production by TLR-activated CLL cells and in some cases even increased it (**Supplementary Figure 6C**). *IL10* expression is regulated by STAT3 (37) and a number of cytokines contribute to autocrine activation of STAT3 in TLR-activated CLL cells that would be blocked by ruxolitinib (**Figure 2E**). These observations suggested IL-10 might auto-regulate its own production in concert with cytokines such as IL-6 and -2. Consistent with this idea, *IL10* transcripts in resting CLL cells were increased in a patient-specific manner after 24 h by exogenous IL-10 but combinations of IL-6, -2, and -10 produced higher expression (**Figure 5A**). *IL10* transcripts in 2S cells were also reduced strongly by anti-IL10 and -IL10 receptor neutralizing antibodies (**Figure 5B**).

### Effect of autocrine IL-10 on TLR-responses of normal human B cells

The above results suggested CLL cells activated by IL-2 and a TLR7-agonist in the presence of ruxolitinib cannot make high amounts of IL-10 due to the absence of an amplifying signal from cytokines including IL-10 but also exhibit signs of increased NFκB-signaling (**Figure 3**). An insight into the functional implications of low IL-10 production by TLR-activated B cells was provided by the database of Heine et al. (45). In this study, purified normal human B cells were sorted on the basis of IL-10 production 48 h after stimulation with a TLR9-agonist along with CD40L and IL-4. Consistent with the observations made with resiquimod in CLL-B cells (**Figures 1** and **3**), analysis of this database (GSE49853) by GSEA suggested TLR-activated B cells that do not make IL-10 exhibit much stronger NFκB activity (**Figure 5C**).

### Effect of ibrutinib on IL-10 and TNFα production by CLL cells

Ibrutinib is also reported to lower IL-10 production by activated CLL cells (11) but partially inhibits TLR-mediated NFκB activation (30). As with ruxolitinib, IL-10 levels in culture supernatants that were increased considerably by IL-2 and resiquimod were lowered dramatically by ibrutinib at 300 nM to mimic plasma levels achieved with conventional dosing *in vivo* (46) (**Figure 5D**, left panel). Low amounts of autocrine IL-10 made spontaneously by CLL cells *in vitro* were also reduced by ibrutinib (**Figure 5D**, left panel). In contrast to JAK inhibition with ruxolitinib (**Figures 3A, C**), early *IL10* transcription (**Figure 5E**) and TNFα, released spontaneously from cultured CLL cells and increased by IL-2 and resiquimod, were both decreased significantly by ibrutinib (**Figure 5D**, right panel). Inhibition of *IL10* mRNA transcription by ibrutinib was maintained at 24 h (**Figure 5F**). These results suggest ibrutinib blocks early induction (within 4 h) of *IL10* mRNA by a TLR-agonist in CLL cells while ruxolitinib blocks late transcription (at 24 h) by inhibiting JAK-activity. Enhanced NFκB activity with ibrutinib is not seen despite lowered IL-10 presumably because of simultaneous partial inhibition of TLR-signaling (30).

## Discussion

The results in this paper suggest: 1. JAK-activating cytokines, particularly IL-10, increase STAT3-phosphorylation and inhibit subsequent TLR-signaling in CLL cells (**Figures 2**, **4**). 2. Ruxolitinib prevents STAT3-phosphorylation (**Figure 2D**) while increasing IRAK4/NFκB/p38-signaling in CLL cells *in vitro* (**Figures 2B** and **3B**) and *in vivo* (**Figure 1**) (13). 3. Both ibrutinib and ruxolitinib block IL-10 production by TLR-activated CLL cells but ruxolitinib prevents sustained transcription and translation of *IL10* mRNA (**Figures 2**, **3**) while ibrutinib blocks early transcription (**Figure 5**).

An aberrant cytokine network is associated with CLL (47). Our clinical observations that non-specific JAK inhibition with ruxolitinib causes CLL cells to become more activated (**Figure 1**) (13–15) suggest this network may arise to suppress the activation of CLL cells in order to prevent disease progression. CLL cells also become more activated even when ruxolitinib is administered in the presence of ibrutinib (14, 15). This observation suggests ongoing suppressive cytokine activity may contribute to the therapeutic activity of ibrutinib and possibly other BTK-inhibitors.

**Figure 6:**
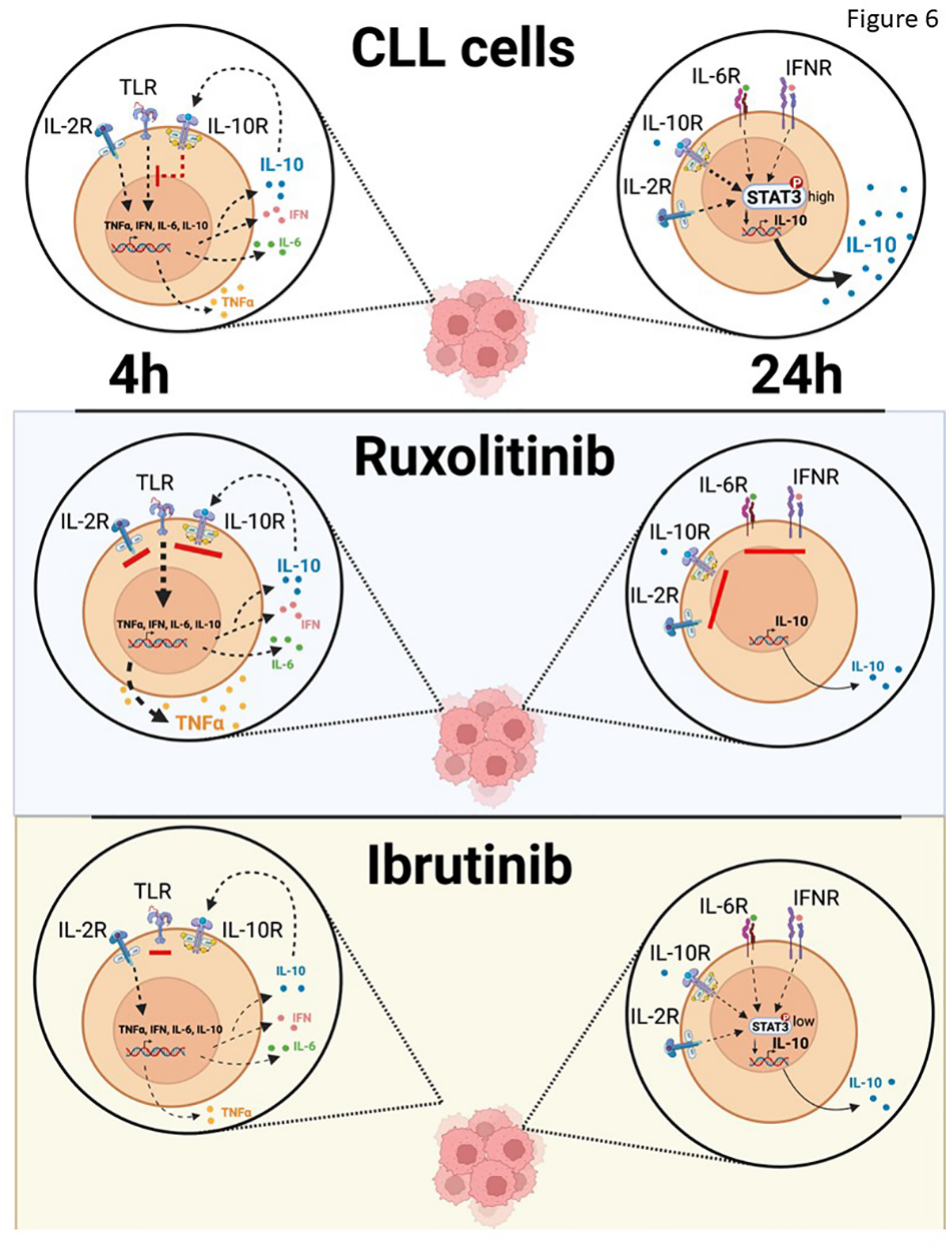
Schema of effect of IL10 on TLR-signaling *in vivo*. IL-10 sustained in part by IL-6 or possibly IFN suppresses TLR-7-signaling in CLL cells, which is unleashed by ruxolitinib to make NFκB-regulated cytokines such as TNFα. Ibrutinib also blocks IL-10 production by CLL cells but prevents their activation by partially inhibiting the NFκB pathway directly and remaining susceptible to paracrine and endocrine IL10 from sources other than CLL cells.

What are the activating signals for CLL cells that are being repressed by cytokines? Signals from the BCR are generally considered the main drivers of CLL cell proliferation *in vivo* (1, 48) but it seems unlikely that constraints on BCR-signaling are being released by ruxolitinib as cytokines like IL-4 promote rather than inhibit this pathway in CLL cells (**Figure 4**) (8). Other pathways that activate NFκB and p38 and are associated with TNF-signaling (**Figure 1**) appear to be more affected by ruxolitinib. Extrapolation of the *in vitro* evidence presented in this manuscript suggests IRAK4-activating signals (**Figure 2B**), possibly from TLRs, may be under chronic inhibition by the cytokine network that is relieved by ruxolitinib (**Figure 6**). Some of these signals may still be active and being repressed by ongoing cytokine activity in the presence of ibrutinib, explaining why a similar activation phenotype was seen in CLL cells following treatment of patients on ibrutinib with ruxolitinib (13–15). While release of TLR-signaling from JAK-mediated inhibition is a plausible explanation for the clinical observations with ruxolitinib (13–15), other JAK-activating factors than the ones studied in this paper and drivers of CLL progression such as wnt and Notch-signaling could potentially also play a role and require further study.

What are the specific repressive cytokines that are susceptible to ruxolitinib *in vivo*? IL-4 inhibits TLR-signaling, which would be reversed by ruxolitinib, but it also enhances BCR-signaling (**Figure 4**), suggesting CLL cells should become less activated *in vivo* if ruxolitinib was mainly blocking the effects of IL-4. Clinical responses should have also been observed if ruxolitinib was mainly blocking the growth-promoting activity of IFN-signaling (9, 10). JAK inhibitors would also inhibit IL-6 and CXCR4-signaling but these pathways appear to have relatively weak effects on TLRs in CLL cells (**Figure 4A**). IL-10 might be the major target of ruxolitinib as it is a strong inhibitor of TLR-signaling (**Figure 4A**) and loss of autocrine IL-10 activity is associated with enhanced TLR-responses of normal B cells (**Figure 5C**). Interestingly, CLL cells that exhibit more aggressive clinical behavior make less IL-10 and respond more strongly to TLR-signals (26, 49).

If ruxolitinib is decreasing IL10 transcription, translation, and signaling *in vivo*, why do circulating CLL cells express high levels of IL-10 signature genes such as *IL10* itself and *SOCS3* (**Figure 1B** and **Supplementary Figure 1A**) (37)? Both are also regulated by NFκB (50, 51) and their increased expression is likely due to enhanced NFκB activity as seen early after activation of CLL cells with IL2 and resiquimod *in vitro* (**Figure 3A**). But then where are the *in vivo* equivalents of the cells that turn off *IL10* after 24 h in the presence of ruxolitinib (**Figure 3A**)? It seems unlikely they have died off, as ruxolitinib was essentially non-toxic to CLL cells *in vitro* (**Supplementary Figure 3**). A major feature of ruxolitinib in CLL patients *in vivo* is altered migration of leukemia cells (13–15). Perhaps changes in chemokine and homing receptors that may accompany down-regulation of *IL10* trap CLL cells in extravascular environments so that only recently activated CLL cells are present in the blood.

Ibrutinib (52) and ruxolitinib (**Figure 1E**) both decrease IL-10 in CLL patients but, unlike ruxolitinib, ibrutinib deactivates CLL cells *in vivo* (16). If IL-10 acts like a tumor suppressor in CLL, why does ibrutinib not also activate CLL cells? Ruxolitinib does not affect initial *IL10* induction and even enhances it (**Figure 3A**) but blocks the effects of intermediate cytokines like IL-2, IL-6, and IL-10 that maintain *IL10* transcription and translation (**Figures 2E**, **5A, B**). As IL-10 inhibits TLR-responses, the result is that TLR-signaling is enhanced by ruxolitinib (**Figure 6**). In contrast, ibrutinib partially blocks TLR-signaling (30) to inhibit early *IL10* gene expression (**Figures 5E, F**), preventing amplification of TLR-signaling and markedly decreasing the activation state of CLL cells (**Figure 6**). Moreover, paracrine or endocrine IL-10 could replace loss of autocrine IL-10 from ibrutinib *in vivo* but not from ruxolitinib that would continue to block IL-10 signaling from exogenous sources. These different mechanisms may relate to the diametrically opposed clinical outcomes of ruxolitinib and ibrutinib.

Ruxolitinib has therapeutic activity in cancers like myelofibrosis driven primarily by oncogenic JAK-signaling (53). Our results suggest JAK inhibitors should be used with caution in cancers driven by pathogenic activation of both NFκB and STAT3 such as CLL (50, 51). By blocking the effects of inhibitory cytokines like IL-10, ruxolitinib may promote NFκB-signaling and perhaps even tumor progression (54). This mechanism may help explain the apparent increased risk of aggressive B cell lymphomas in myelofibrosis patients on ruxolitinib (55). Specific inhibitors of growth-promoting cytokines such as IFNAR antibodies (10, 18) or infusing suppressive cytokines such as IL-10 might be better strategies than non-specific inhibition with JAK inhibitors for therapeutic manipulation of cytokines in CLL.

## Data availability statement

The datasets presented in this study can be found in online repositories. The names of the repository/repositories and accession number(s) can be found below: https://www.ncbi.nlm.nih.gov/geo/, GSE197811.

## Ethics statement

All studies involving human samples were reviewed and approved by the Sunnybrook Research Ethics Board (PIN 222-2014). The patients/participants provided their written informed consent to participate in this study.

## Author contributions

DS conceived the project and wrote the manuscript. TL, YS, and GW obtained CLL cells and performed cell culture, RT-PCR, and immunoblotting. DH and GS contributed to GSEA analyses. All the authors reviewed versions of the manuscript and approved the final version. DS had final responsibility for the decision to submit the manuscript for publication. All authors contributed to the article and approved the submitted version.

## References

[1] Barr PM Owen C Robak T Tedeschi A Bairey O Burger JA. Up to 8-year follow-up from RESONATE-2: first-line ibrutinib treatment for patients with chronic lymphocytic leukemia. Blood Adv (2022) 6(11):3440–50. doi: 10.1182/bloodadvances.2021006434 PMC9198904 35377947

[2] Ma S Seymour JF Brander DM Kipps TJ Choi MY Anderson MA. Efficacy of venetoclax plus rituximab for relapsed CLL: 5-year follow-up of continuous or limited- duration therapy. Blood (2021) 138(10):836–46. doi: 10.1182/blood.2020009578 PMC9710452 34115103

[3] Kochenderfer JN Dudley ME Kassim SH Somerville RP Carpenter RO Stetler-Stevenson M. Chemotherapy-refractory diffuse large B-cell lymphoma and indolent B-cell malignancies can be effectively treated with autologous T cells expressing an anti-CD19 chimeric antigen receptor. J Clin Oncol (2015) 33(6):540–9. doi: 10.1200/JCO.2014.56.2025 PMC4322257 25154820

[4] Hu X Li J Fu M Zhao X Wang W. The JAK/STAT signaling pathway: From bench to clinic. Signal Transduct Target Ther (2021) 6(1):402. doi: 10.1038/s41392-021-00791-1 34824210 PMC8617206

[5] Severin F Frezzato F Visentin A Martini V Trimarco V Carraro S. In chronic lymphocytic leukemia the JAK2/STAT3 pathway is constitutively activated and its inhibition leads to CLL cell death unaffected by the protective bone marrow microenvironment. Cancers (Basel) (2019) 11(12):1939. doi: 10.3390/cancers11121939 31817171 PMC6966457

[6] Tomic J Lichty B Spaner DE. Aberrant interferon-signaling is associated with aggressive chronic lymphocytic leukemia. Blood (2011) 117(9):2668–80. doi: 10.1182/blood-2010-05-285999 21205928

[7] Burger JA Montserrat E. Coming full circle: 70 years of chronic lymphocytic leukemia cell redistribution, from glucocorticoids to inhibitors of BS cell receptor signaling. Blood (2013) 121(9):1501–9. doi: 10.1182/blood-2012-08-452607 PMC4968370 23264597

[8] Steele AJ Prentice AG Cwynarski K Hoffbrand AV Hart SM Lowdell MW. The JAK3-selective inhibitor PF-956980 reverses the resistance to cytotoxic agents induced by interleukin-4 treatment of chronic lymphocytic leukemia cells: Potential for reversal of cytoprotection by the microenvironment. Blood (2010) 116(22):4569–77. doi: 10.1182/blood-2009-09-245811 20716767

[9] Xia M Luo TY Shi Y Wang G Tsui H Harari D. Effect of ibrutinib on the IFN response of chronic lymphocytic leukemia cells. J Immunol (2020) 205(10):2629–39. doi: 10.4049/jimmunol.2000478 33067379

[10] Luo TY Shi Y Wang G Spaner DE. Enhanced interferon-sensing by aggressive chronic lymphocytic leukemia cells. J Immunol (2022) 209(9):1662–73. doi: 10.4049/jimmunol.2200199 36104109

[11] Long M Beckwith K Do P Mundy BL Gordon A Lehman AM. Ibrutinib treatment improves T cell number and function in CLL patients. J Clin Invest (2017) 127(8):3052–64. doi: 10.1172/JCI89756 PMC5531425 28714866

[12] Rivas JR Liu Y Alhakeem SS Eckenrode JM Marti F Collard JP. Interleukin-10 suppression enhances T-cell antitumor immunity and responses to checkpoint blockade in chronic lymphocytic leukemia. Leukemia (2021) 35(11):3188–200. doi: 10.1038/s41375-021-01217-1 PMC8446094 33731852

[13] Spaner DE Wang G McCaw L Li Y Disperati P Cussen MA. Activity of the janus kinase inhibitor ruxolitinib in chronic lymphocytic leukemia: Results of a phase II trial. Haematologica (2016) 101(5):e192–5. doi: 10.3324/haematol.2015.135418 PMC5004376 26819050

[14] Spaner DE McCaw L Wang G Tsui H Shi Y. Persistent janus kinase-signaling in chronic lymphocytic leukemia patients on ibrutinib: Results of a phase I trial. Cancer Med (2019) 8(4):1540–50. doi: 10.1002/cam4.2042 PMC6488147 30843659

[15] Spaner DE Luo Y Wang G Gallagher J Tsui H Shi Y. Janus kinases restrain chronic lymphocytic leukemia cells in patients on ibrutinib: Results of a phase II trial. Cancer Med (2021) 10(24):8789–98. doi: 10.1002/cam4.4378 PMC8683523 34791813

[16] Landau DA Sun C Rosebrock D Herman SEM Fein J Sivina M. The evolutionary landscape of chronic lymphocytic leukemia treated with ibrutinib targeted therapy. Nat Commun (2017) 8(1):2185. doi: 10.1038/s41467-017-02329-y 29259203 PMC5736707

[17] Johnson CS Cook CA Furmanski P. *In vivo* suppression of erythropoiesis by tumor necrosis factor-alpha (TNF-alpha): Reversal with exogenous erythropoietin (EPO). Exp Hematol (1990) 18(2):109–13. 2303102

[18] Riggs JM Hanna RN Rajan B Zerrouki K Karnell JL Sagar D. Characterization of anifrolumab, a fully human anti-interferon receptor antagonist antibody for the treatment of systemic lupus erythematosus. Lupus Sci Med (2018) 5(1):e000261. doi: 10.1136/lupus-2018-000261 29644082 PMC5890856

[19] Shi Y Wang G Muhowski EM McCaw L Wang C Bjarnason G. Ibrutinib reprograms the glucocorticoid receptor in chronic lymphocytic leukemia cells. Leukemia (2019) 33(7):1650–62. doi: 10.1038/s41375-019-0381-4 30696950

[20] Oppermann S Ylanko J Shi Y Hariharan S Oakes CC Brauer PM. High-content screening identifies kinase inhibitors that overcome venetoclax resistance in activated CLL cells. Blood (2016) 128(7):934–47. doi: 10.1182/blood-2015-12-687814 PMC5000578 27297795

[21] Shi Y White D He L Miller RL Spaner DE. Toll-like receptor-7 tolerizes malignant B cells and enhances killing by cytotoxic agents. Cancer Res (2007) 67(4):1823–31. doi: 10.1158/0008-5472.CAN-06-2381 17308125

[22] Reimand J Isserlin R Voisin V Kucera M Tannus-Lopes C Rostamianfar A. Pathway enrichment analysis and visualization of omics data using g:Profiler, GSEA, cytoscape and EnrichmentMap. Nat Protoc (2019) 14(2):482–517. doi: 10.1038/s41596-018-0103-9 30664679 PMC6607905

[23] Subramanian A Tamayo P Mootha VK Mukherjee S Ebert BL Gillette MA. Gene set enrichment analysis: A knowledge-based approach for interpreting genome-wide expression profiles. Proc Natl Acad Sci U.S.A. (2005) 102(43):15545–50. doi: 10.1073/pnas.0506580102 PMC1239896 16199517

[24] Liberzon A Subramanian A Pinchback R Thorvaldsdóttir H Tamayo P Mesirov JP. Molecular signatures database (MSigDB) 3.0. Bioinformatics (2011) 27(12):1739–40. doi: 10.1093/bioinformatics/btr260 PMC3106198 21546393

[25] Alexander AF Kelsey I Forbes H Miller-Jensen K. Single-cell secretion analysis reveals a dual role for IL-10 in restraining and resolving the TLR4-induced inflammatory response. Cell Rep (2021) 36(12):109728. doi: 10.1016/j.celrep.2021.109728 34551303 PMC8995750

[26] Drennan S D'Avola A Gao Y Weigel C Chrysostomou E Steele AJ. IL-10 production by CLL cells is enhanced in the anergic IGHV mutated subset and associates with reduced DNA methylation of the IL10 locus. Leukemia (2017) 31(8):1686–94. doi: 10.1038/leu.2016.356 27890932

[27] Chen X Shi JG Emm T Scherle PA McGee RF Lo Y. Pharmacokinetics and pharmacodynamics of orally administered ruxolitinib (INCB018424 phosphate) in renal and hepatic impairment patients. Clin Pharmacol Drug Dev (2014) 3(1):34–42. doi: 10.1002/cpdd.77 27128228

[28] Giménez N Schulz R Higashi M Aymerich M Villamor N Delgado J. Targeting IRAK4 disrupts inflammatory pathways and delays tumor development in chronic lymphocytic leukemia. Leukemia (2020) 34(1):100–14. doi: 10.1038/s41375-019-0507-8 PMC8075947 31197259

[29] Mitchell K Barreyro L Todorova TI Taylor SJ Antony-Debré I Narayanagari S-R. IL1RAP potentiates multiple oncogenic signaling pathways in AML. J Exp Med (2018) 215:170–27. doi: 10.1084/jem.20180147 PMC5987926 29773641

[30] Dadashian EL McAuley EM Liu D Shaffer AL 3rd Young RM Iyer JR. TLR signaling is activated in lymph node-resident CLL cells and is only partially inhibited by ibrutinib. Cancer Res (2019) 79(2):360–71. doi: 10.1158/0008-5472.CAN-18-0781 PMC6342512 30498085

[31] Herishanu Y Pérez-Galán P Liu D Biancotto A Pittaluga S Vire B. The lymph node microenvironment promotes B-cell receptor signaling, NFκB activation, and tumor proliferation in chronic lymphocytic leukemia. Blood (2011) 117(2):563–74. doi: 10.1182/blood-2010-05-284984 PMC3031480 20940416

[32] Brander D Islam P Barrientos JC. Tailored treatment strategies for chronic lymphocytic leukemia in a rapidly changing era. Am Soc Clin Oncol Educ Book (2019) 39:487–98. doi: 10.1200/EDBK_238735 31099686

[33] Alhakeem SS McKenna MK Oben KZ Noothi SK Rivas JR Hildebrandt GC. Chronic lymphocytic leukemia-derived IL-10 suppresses antitumor immunity. J Immunol (2018) 200(12):4180–9. doi: 10.4049/jimmunol.1800241 PMC6555426 29712773

[34] Li Y Shi Y McCaw L Li YJ Zhu F Gorczynski R. Microenvironmental interleukin-6 suppresses toll-like receptor signaling in human leukemia cells through miR-17/19A. Blood (2015) 126(6):766–78. doi: 10.1182/blood-2014-12-618678 26041742

[35] Tomic J White D Shi Y Mena J Hammond C He L. Sensitization of IL-2 signaling through TLR-7 enhances B lymphoma cell immunogenicity. J Immunol (2006) 176(6):3830–9. doi: 10.4049/jimmunol.176.6.3830 16517754

[36] Deimel LP Li Z Roy S Ranasinghe C. STAT3 determines IL-4 signaling outcomes in naïve T cells. Sci Rep (2021) 11(1):10495. doi: 10.1038/s41598-021-89860-7 34006897 PMC8131749

[37] Saraiva M Vieira P O'Garra A. Biology and therapeutic potential of interleukin-10. J Exp Med (2020) 217(1):e20190418. doi: 10.1084/jem.20190418 31611251 PMC7037253

[38] Spaner DE Shi Y White D Mena J Hammond C Tomic J. Immunomodulatory effects of toll-like receptor-7 activation on chronic lymphocytic leukemia cells. Leukemia (2006) 20(2):286–95. doi: 10.1038/sj.leu.2404061 16341037

[39] Bennett BL Cruz R Lacson RG Manning AM. Interleukin-4 suppression of tumor necrosis factor alpha-stimulated e-selectin gene transcription is mediated by STAT6 antagonism of NFκB. J Biol Chem (1997) 272(15):10212–9. doi: 10.1074/jbc.272.15.10212 9092569

[40] Wolf MJ Hoos A Bauer J Boettcher S Knust M Weber A. Endothelial CCR2 signaling induced by colon carcinoma cells enables extravasation *via* the JAK2-Stat5 and p38MAPK pathway. Cancer Cell (2012) 22(1):91–105. doi: 10.1016/j.ccr.2012.05.023 22789541

[41] Mueller A Strange PG. CCL3, acting via the chemokine receptor CCR5, leads to independent activation of janus kinase 2 (JAK2) and gi proteins. FEBS Lett (2004) 570(1-3):126–32. doi: 10.1016/j.febslet.2004.04.100 15251452

[42] Aguilar-Hernandez MM Blunt MD Dobson R Yeomans A Thirdborough S Larrayoz M. IL-4 enhances expression and function of surface IgM in CLL cells. Blood (2016) 127:3015–25. doi: 10.1182/blood-2015-11-682906 27002119

[43] Martines C Chakraborty S Vujovikj M Gobessi S Vaisitti T Deaglio S. Macrophage-and BCR-derived but not TLR-derived signals support the growth of CLL and Richter syndrome murine models *in vivo* . Blood (2022) 140(22):2335–47. doi: 10.1182/blood.2022016272 36084319

[44] Pattison MJ Mackenzie KF Arthur JS. Inhibition of JAKs in macrophages increases lipopolysaccharide-induced cytokine production by blocking IL-10-mediated feedback. J Immunol (2012) 189(6):2784–92. doi: 10.4049/jimmunol.1200310 PMC3443740 22904308

[45] Heine G Drozdenko G Grün JR Chang HD Radbruch A Worm M. Autocrine IL-10 promotes human B-cell differentiation into IgM- or IgG-secreting plasmablasts. Eur J Immunol (2014) 44(6):1615–21. doi: 10.1002/eji.201343822 24643722

[46] Advani RH Buggy JJ Sharman JP Smith SM Boyd TE Grant B. Bruton tyrosine kinase inhibitor ibrutinib (PCI-32765) has significant activity in patients with relapsed/refractory B-cell malignancies. J Clin Oncol (2013) 31(1):88–94. doi: 10.1200/JCO.2012.42.7906 23045577 PMC5505166

[47] Yan XJ Dozmorov I Li W Yancopoulos S Sison C Centola M. Identification of outcome-correlated cytokine clusters in chronic lymphocytic leukemia. Blood (2011) 118(19):5201–10. doi: 10.1182/blood-2011-03-342436 PMC3217404 21911837

[48] Burger JA Chiorazzi N. B cell receptor signaling in chronic lymphocytic leukemia. Trends Immunol (2013) 34(12):592–601. doi: 10.1016/j.it.2013.07.002 23928062 PMC3898793

[49] Tarnani M Laurenti L Longo PG Piccirillo N Gobessi S Mannocci A. The proliferative response to CpG-ODN stimulation predicts PFS, TTT and OS in patients with chronic lymphocytic leukemia. Leuk Res (2010) 34(9):1189–94. doi: 10.1016/j.leukres.2009.12.020 20074801

[50] Grivennikov SI Karin M. Dangerous liaisons: STAT3 and NF-kappaB collaboration and crosstalk in cancer. Cytokine Growth Factor Rev (2010) 21(1):11–9. doi: 10.1016/j.cytogfr.2009.11.005 PMC2834864 20018552

[51] Liu FT Jia L Wang P Wang H Farren TW Agrawal SG. STAT3 and NF-κB cooperatively control *in vitro* spontaneous apoptosis and poor chemo-responsiveness in patients with chronic lymphocytic leukemia. Oncotarget (2016) 7(22):32031–45. doi: 10.18632/oncotarget.8672 PMC5077994 27074565

[52] Niemann CU Herman SE Maric I Gomez-Rodriguez J Biancotto A Chang BY. Disruption of *in vivo* chronic lymphocytic leukemia tumor-microenvironment interactions by ibrutinib–findings from an investigator-initiated phase II study. Clin Cancer Res (2016) 22(7):1572–82. doi: 10.1158/1078-0432.CCR-15-1965 PMC4818677 26660519

[53] Harrison CN Vannucchi AM Kiladjian JJ Al-Ali HK Gisslinger H Knoops L. Long-term findings from COMFORT-II, a phase 3 study of ruxolitinib vs best available therapy for myelofibrosis. Leukemia (2017) 31(3):775. doi: 10.1038/leu.2016.323 28248313 PMC7609269

[54] Grabner B Schramek D Mueller KM Moll HP Svinka J Hoffmann T. Disruption of STAT3 signalling promotes KRAS-induced lung tumorigenesis. Nat Commun (2015) 6:6285. doi: 10.1038/ncomms7285 25734337 PMC4366489

[55] Porpaczy E Tripolt S Hoelbl-Kovacic A Gisslinger B Bago-Horvath Z Casanova-Hevia E. Aggressive B-cell lymphomas in patients with myelofibrosis receiving JAK1/2 inhibitor therapy. Blood (2018) 132(7):694–706. doi: 10.1182/blood-2017-10-810739 29907599 PMC7115916

