# Supplementary material for: Paradoxical activation of chronic lymphocytic leukemia cells by ruxolitinib in vitro and in vivo
Source: Front Oncol. 2023 Apr 11;13:1043694. doi: 10.3389/fonc.2023.1043694 (PMC10126367; doi:10.3389/fonc.2023.1043694)
Supplement: Supplementary file 1 [file DataSheet_1.pdf]

Supplementary figures 1-6

A

	SYMBOL	RANK IN GENE LIST	RANK METRIC SCORE	RUNNING ES
153	MYC	12388	-0.658	-0.3034
154	AREG	12390	-0.659	-0.2933
155	PDE4B	12395	-0.661	-0.2833
156	PER1	12402	-0.669	-0.2734
157	GPR183	12405	-0.672	-0.2632
158	DUSP1	12431	-0.695	-0.2543
159	CD83	12458	-0.720	-0.2452
160	MAFF	12501	-0.761	-0.2367
161	DUSP2	12511	-0.771	-0.2255
162	IRS2	12524	-0.785	-0.2142
163	PTGER4	12582	-0.846	-0.2056
164	TRIB1	12653	-0.929	-0.1967
165	EGR2	12656	-0.930	-0.1825
166	EGR1	12657	-0.930	-0.1680
167	DUSP5	12703	-1.032	-0.1556
168	FOS	12741	-1.166	-0.1404
169	CCL4	12750	-1.189	-0.1226
170	SOCS3	12757	-1.203	-0.1044
171	NR4A2	12779	-1.319	-0.0856
172	FOSL2	12795	-1.512	-0.0633
173	NR4A3	12802	-1.714	-0.0372
174	DUSP4	12807	-2.418	-0.0000

B

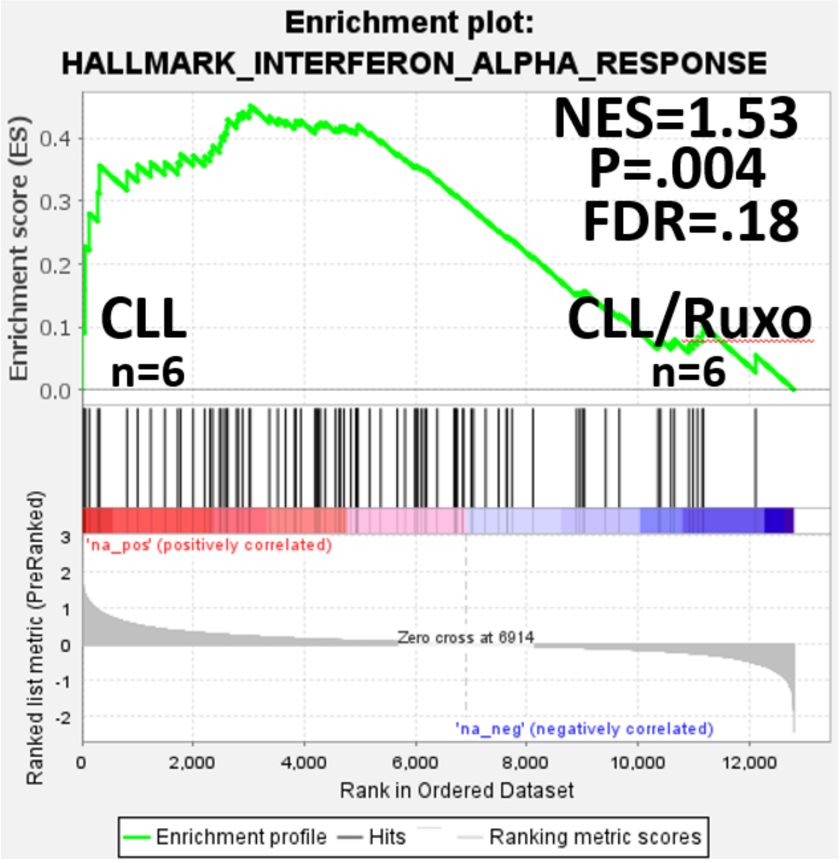

Supplementary figure 1: Effect of ruxolitinib on NFκB- and interferon-regulated genes *in vivo*. Differentially expressed genes in CLL cells purified before and after administration of ruxolitinib were analyzed by GSEA. **A.** Core enrichment genes for the "Hallmark_TNFA_Signaling_via_NFκB" data set are shown. **B.** The enrichment plot indicates down-regulation of interferon-alpha response genes in CLL cells exposed to ruxolitinib *in vivo*.

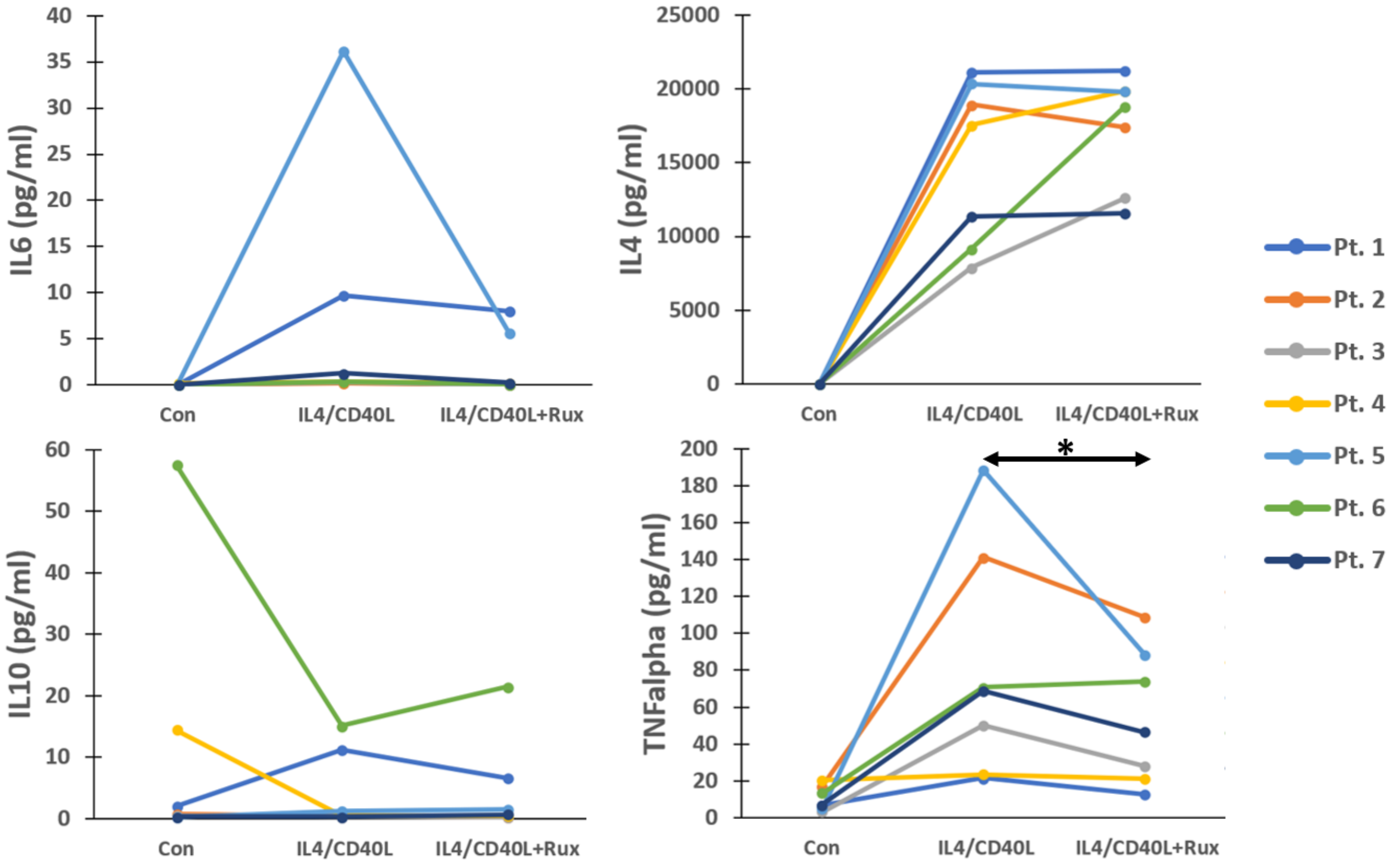

Supplementary figure 2. Effect of ruxolitinib on cytokine production by CLL cells in the IL4/CD40L microenvironmental model. CLL cells from 7 different patients were cultured on OP9 stromal cells (Con) or on OP9 cells engineered to express CD40L with IL-4 (10ng/ml) with or without ruxolitinib (500 nM) as described in the materials and methods. After 48 h, IL4, IL10, IL6, and TNFα levels were measured in the culture supernatants. Each line indicates the results from an individual patient. IL4 is confirmed to be present in the activation cultures. Unlike CLL cells treated with IL2 and resiquimod, TNF levels were significantly decreased by ruxolitinib while IL10 was both inconsistently induced and affected by ruxolitinib. *, $p<0.05$

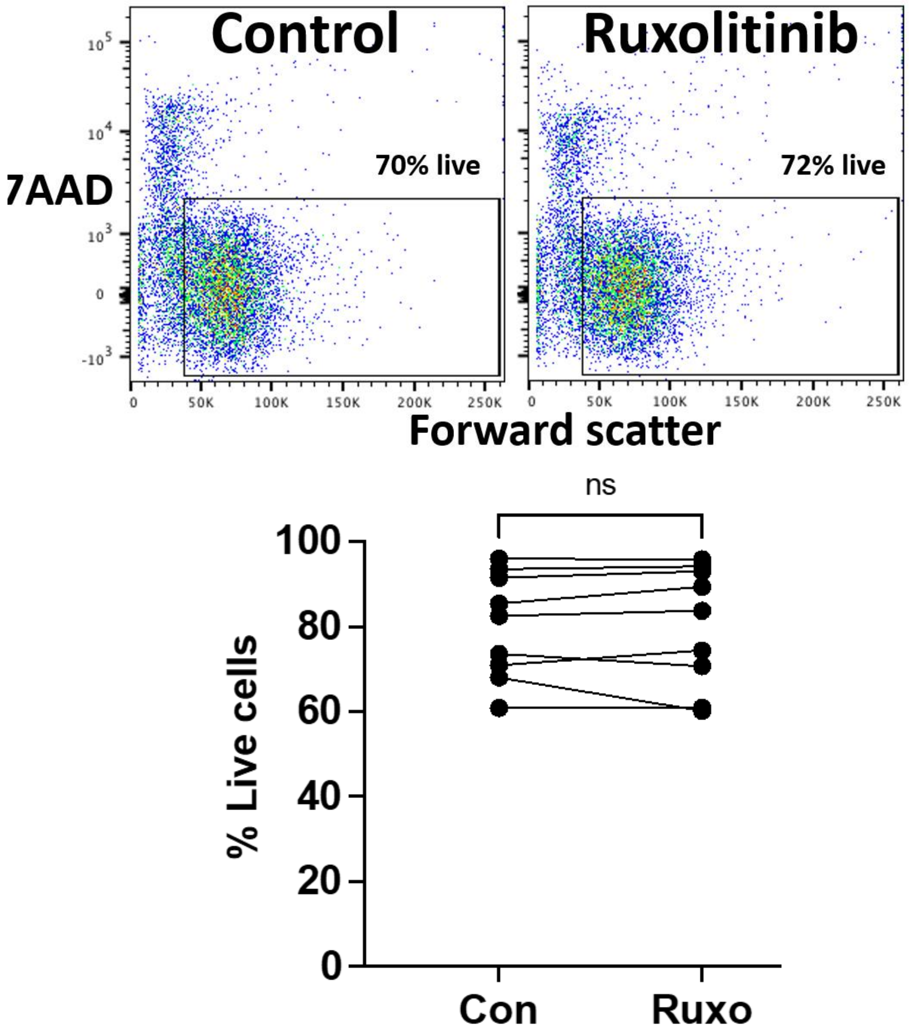

Supplementary figure 3: Viability of CLL cells in the presence of ruxolitinib. CLL cells were cultured in AIM-V media in the presence and absence of ruxolitinib (500 nM). Viable cells after 48 h were indicated by the percentage that excluded the nuclear dye 7AAD as measured by flow cytometry. An example is shown and results for 9 different patients are shown in the summary graph with each line representing results for an individual sample. The results indicate ruxolitinib was not toxic to CLL cells in these serum-free conditions.

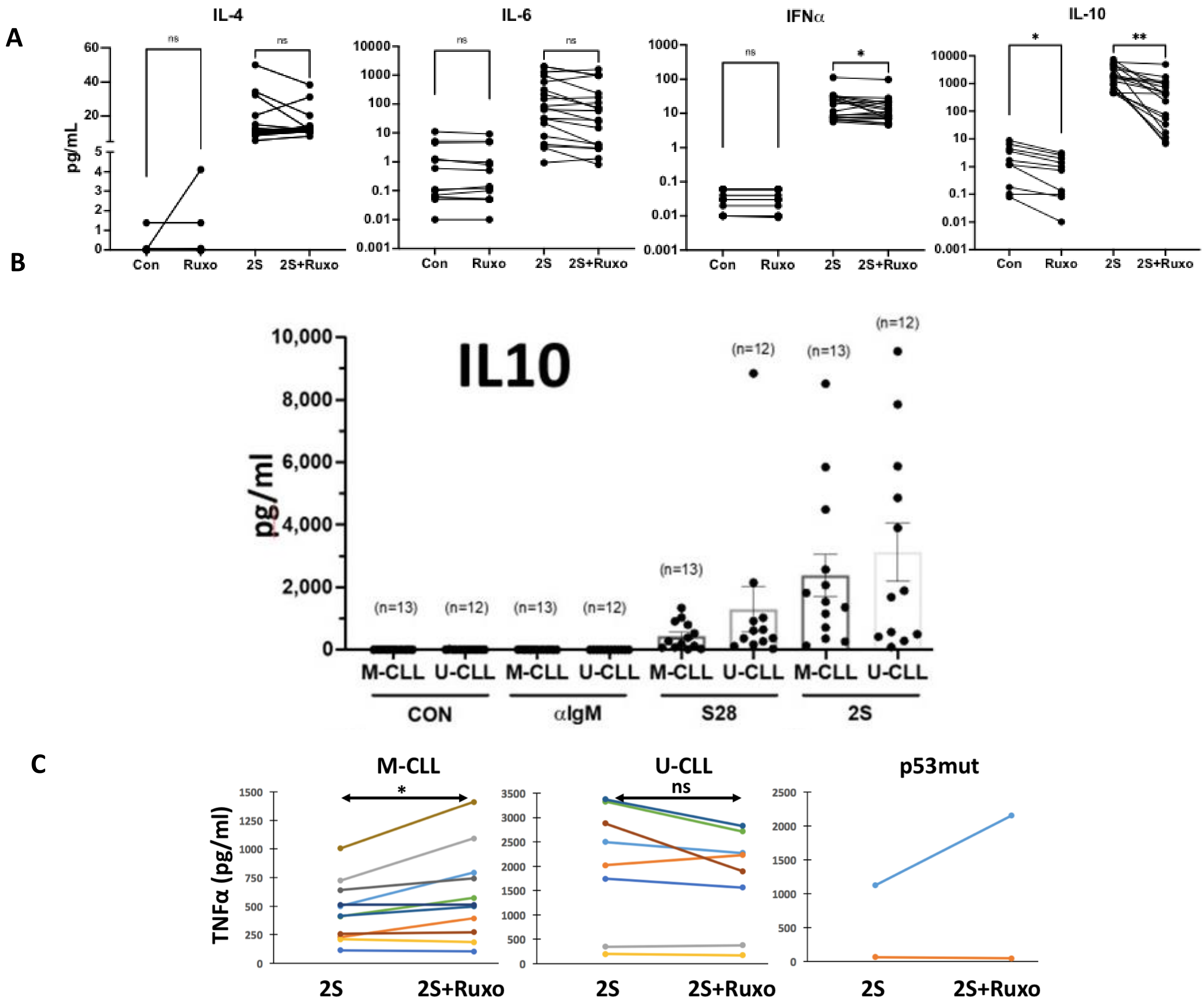

Supplementary figure 4. Effect of ruxolitinib on cytokine production from individual CLL samples. CLL cells from different patients were cultured with resiquimod (S28), with or without IL2 and resiquimod (2S), or activated with anti-IgM antibodies (10 ng/mL) in the presence and absence of ruxolitinib. Cytokines in culture supernatants were then measured after 48 h. **A.** The effect of ruxolitinib on IL-4, -6, -10, and IFNα production by CLL cells cultured directly *ex vivo* with or without 2S activation are shown for individual patients. **B.** IL10 production is shown for individual patient samples in the different activation conditions without ruxolitinib, grouped according to expression of mutated (M-CLL) or unmutated (U-CLL) *IGHV* genes. **C.** TNFα was measured after 48 h for 21 patients. The results are shown for 11 M-CLL, 8 U-CLL, and 2 patients with inactivating p53 mutations. Each line represents a different sample. *, p<.05; **, p<.001; ns=not significant. Statistical analysis was done with students paired t tests.

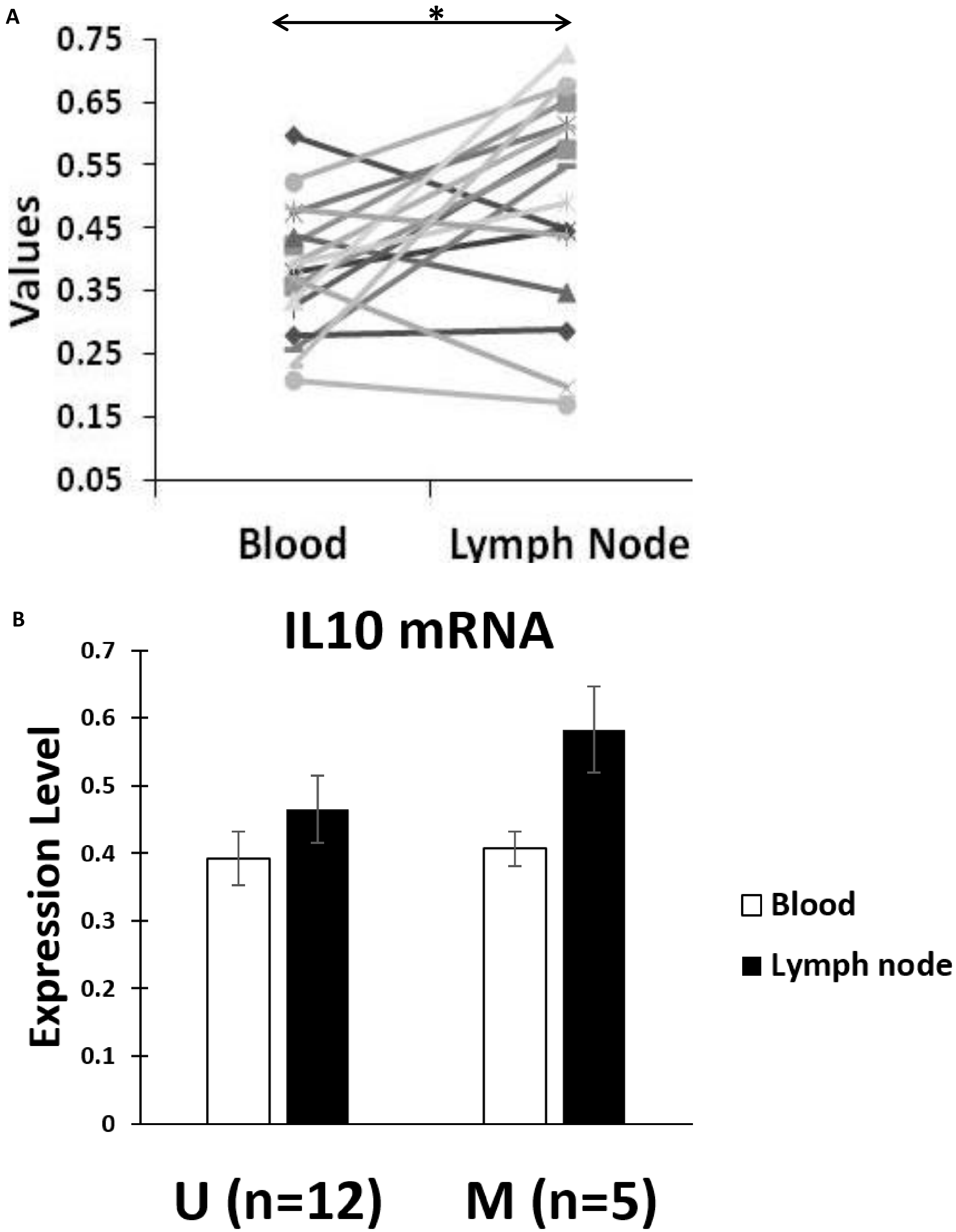

Supplementary figure 5. IL10 mRNA expression in blood and lymph nodes of CLL patients. A. *In silico* analysis of GEO database GSE21029 composed of genes expressed in paired CLL cells from lymph nodes and blood of 17 patients using the data analysis tool "find genes" suggests *IL10* transcription increases when circulating CLL cells enter lymph node microenvironments. Each line represents results for a single patient. **B.** Table 1 of the publication that described the database indicated 12 patients had CLL cells with unmutated *IGHV* genes (U) and 5 had mutated *IGHV* genes (M). Expression levels of *IL10* for each sample were extracted manually from the table of values that accompanied the graph on the GEO profiles web site. Averages and standard errors of the results for mutated and unmutated blood and lymph node samples are shown and indicate a trend toward higher *IL10* mRNA expression in mutated *IGHV* samples in both blood and lymph node microenvironments. *, p<.05

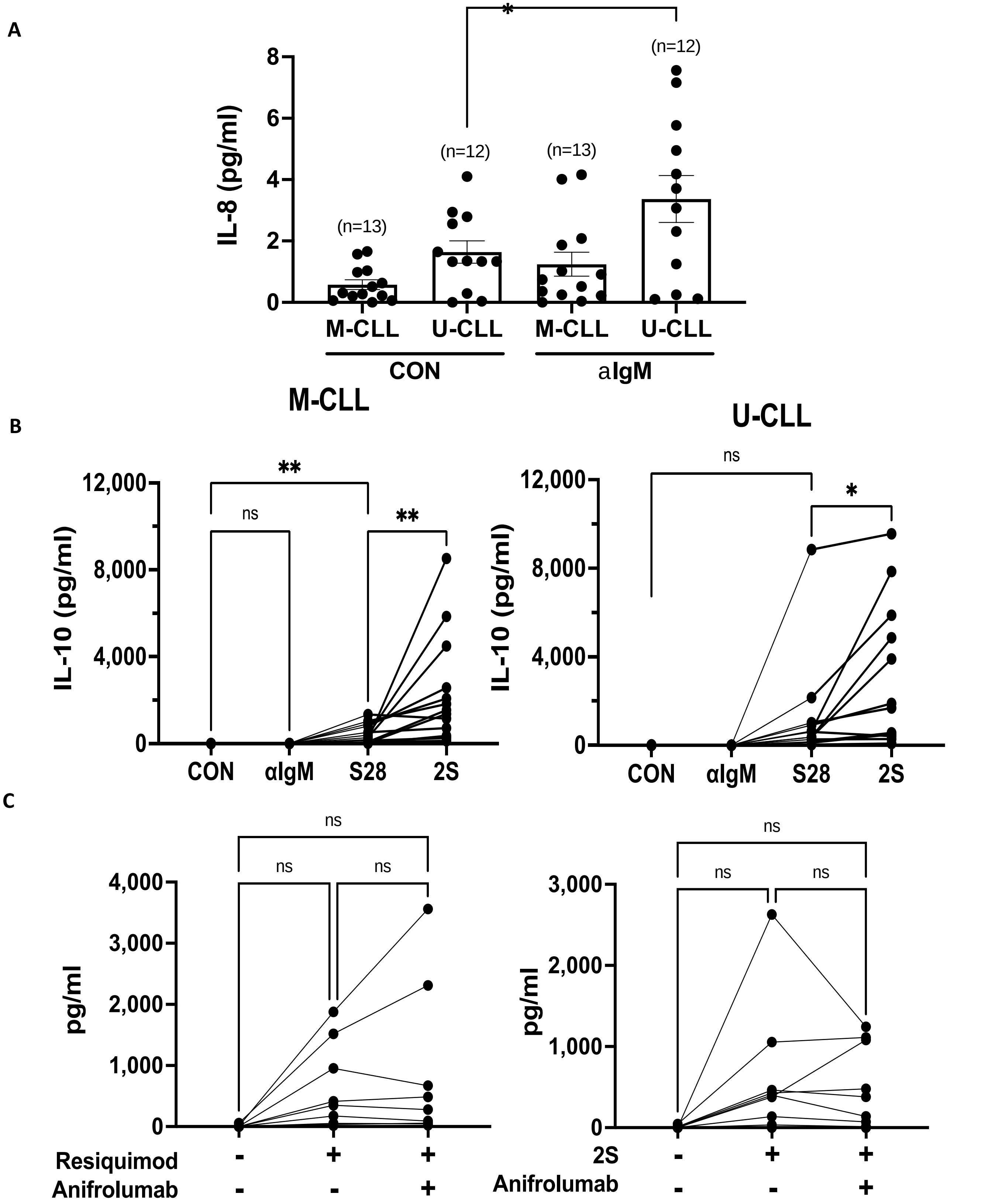

Supplementary figure 6. Effect of *IGHV* mutation status and IFNAR antibodies on IL-8 and IL-10 production by CLL cells. Purified CLL cells were cultured in AIM-V alone (Con) or activated with α-IgM antibodies (10ng/mL), resiquimod (S) (1 μg/ml), or IL2 (500 U/ml) and resiquimod (2S). IL-8 (**A**) and IL10 (**B**) were measured in culture supernatants after 48 h. Averages and standard errors for the numbers of patient samples are indicated. **C.** IL-10 in culture supernatants from 2S (right) or resiquimod (S28)-treated (left) cells activated with or without the anti-IFNAR antibody anifrolumab was measured after 48 h. Averages and standard errors are shown for the indicated numbers of patient samples. The lines represent individual patient samples. *, p<.05; **, p<.01; ns= not significant. Statistical analysis was done by students paired t tests and 2-way ANOVA.
